# Investigating the Potential Contribution of Patient Rating Sites to Hospital Supervision: Exploratory Results From an Interview Study in the Netherlands

**DOI:** 10.2196/jmir.5552

**Published:** 2016-07-20

**Authors:** Sophia Martine Kleefstra, Linda C Zandbelt, Ine Borghans, Hanneke J.C.J.M de Haes, Rudolf B Kool

**Affiliations:** ^1^ Dutch Health Care Inspectorate Department of Risk Detection and Development Utrecht Netherlands; ^2^ Academic Medical Centre Department Medical Psychology University of Amsterdam Amsterdam Netherlands; ^3^ Academic Medical Centre Department od Clinical and Executive Support University of Amsterdam Amsterdam Netherlands; ^4^ Radboud Institute for Health Sciences IQ healthcare Radboud university medical center Nijmegen Netherlands

**Keywords:** patient rating sites, patient satisfaction, patient experiences, hospitals, quality of health care, supervision

## Abstract

**Background:**

Over the last decades, the patient perspective on health care quality has been unconditionally integrated into quality management. For several years now, patient rating sites have been rapidly gaining attention. These offer a new approach toward hearing the patient’s perspective on the quality of health care.

**Objective:**

The aim of our study was to explore whether and how patient reviews of hospitals, as reported on rating sites, have the potential to contribute to health care inspector’s daily supervision of hospital care.

**Methods:**

Given the unexplored nature of the topic, an interview study among hospital inspectors was designed in the Netherlands. We performed 2 rounds of interviews with 10 senior inspectors, addressing their use and their judgment on the relevance of review data from a rating site.

**Results:**

All 10 Dutch senior hospital inspectors participated in this research. The inspectors initially showed some reluctance to use the major patient rating site in their daily supervision. This was mainly because of objections such as worries about how representative they are, subjectivity, and doubts about the relevance of patient reviews for supervision. However, confrontation with, and assessment of, negative reviews by the inspectors resulted in 23% of the reviews being deemed relevant for risk identification. Most inspectors were cautiously positive about the contribution of the reviews to their risk identification.

**Conclusions:**

Patient rating sites may be of value to the risk-based supervision of hospital care carried out by the Health Care Inspectorate. Health care inspectors do have several objections against the use of patient rating sites for daily supervision. However, when they are presented with texts of negative reviews from a hospital under their supervision, it appears that most inspectors consider it as an additional source of information to detect poor quality of care. Still, it should always be accompanied and verified by other quality and safety indicators. More research on the value and usability of patient rating sites in daily hospital supervision and other health settings is needed.

## Introduction

Over the last decades, the patient’s perception of health care quality has been unconditionally integrated into quality management. Traditional patient satisfaction or experience surveys have become accepted tools for measuring health care quality. These tools were demonstrated to add valuable information to professional quality indicators and outcome measures [1,2]. For several years now, a new approach toward hearing the patient’s perspective on the quality of health care, by the use of patient rating sites, has rapidly gained attention. These specialized Internet rating sites allow patients to express and rate their experiences and satisfaction with health care providers and institutions. These ratings are intended to be a source of information on quality for other patients looking for health care providers [3-5]. This is especially the case in the United States, Germany, and the United Kingdom where many patients look for information on these sites. Their use as public reporting instrument is often stimulated by governments [6], supporting patients to make explicit comparisons between health care providers, and hereby increasing public accountability and improving quality of care [7-12].

At first, the introduction of patient rating sites caused doctors and policymakers to raise several objections against the use of this information. They were supposed to be vulnerable to a number of pitfalls, such as being manipulated, showing a large variation in the number of ratings for hospitals and physicians, being emotionally burdensome for physicians who were either criticized or even not rated at all, or being biased by selection of patients, for example, by an overrepresentation of dissatisfied patients [5,8,9,11,13,14]. Furthermore, the average number of ratings for individual physicians was still low, implying that the assessments found for physicians may change over time when more patients took part [8]. Subsequently, rating sites are only used by people who have access to and know how to use the Internet, which could cause bias. Finally, information from rating sites was not case-mix adjusted for patient characteristics such as age, level of education, and health status. This is known to be necessary to prevent bias and thus allow the results to be properly interpreted [15,16].

However, recent results from research on rating sites increasingly questioned these arguments and showed certain advantages. Ratings are mostly positive [4,10,11,17,18] and correlate with relevant clinical outcomes such as decreased mortality, readmissions, infection rates, and decubitus [5,8,19-21]. These correlations are at least as strong as for the traditional paper surveys method [9,19,21]. Moreover, in some cases, the real-time nature of rating sites means that feedback can be given rapidly, which might make the information contained on them more up to date and might thus detect episodes of poor care or outliers in a more timely manner than surveys that took place a long time ago [9]. Also, these ratings can be given to all health care professionals and institutions while survey data regard, mostly, only one part of them. Last but not least, there is reason to believe that these rating sites will become commonplace. In fact, an increasing number of people consult the Internet, looking for health care quality information. This rose from 19% of North American adults in 2001 to 88% in 2010; 24% of them consulted review sites. Also, the number of ratings has risen rapidly. In 2010, up to 16% of all US physicians were reviewed [7,8,10,21], whereas 37% of physicians in Germany were reviewed in 2012 [22]. An awareness of 65% of the US population and a usage of 23% shows that patients are increasingly turning to Web-based rating sites [23]. A German study showed that approximately 65% of patients using a rating site have consulted a particular physician based on these ratings [3]. Thus, despite the arguments against the use of rating sites, these sites do have redeeming value that needs to be further explored.

A recent scoping review concluded that although literature about the topic is still limited, social media, and especially patient rating sites, can become a fast and cheap way to gather information about the quality of care and could complement traditional methods [24]. Thus, although some caution interpreting the information is needed, given methodological restrictions [24], using patient rating sites might help to detect poor performance [9,19,21,25,26]. It is therefore stated that neither physicians nor policy makers should underestimate the growing influence of ratings sites for patients in providing information, and for physicians in offering opportunities to improve the quality of their care, based on the concerns mentioned in reviews [3,4].

Due to the potential value of the information for judging the quality of care, some supervisory bodies already use rating sites as an additional source of information [21,27-30]. In England, for example, the Care Quality Commission actively uses patient rating information from the NHS Choices website, alongside other rating sites, to identify potential risks to patient safety [25,28]. Similar initiatives are found in Australia and Ireland [29].

The Dutch health care Inspectorate’s (IGZ) supervisory framework for risk detection in hospitals contains in the first place several process and outcome indicators developed to monitor the quality and safety of hospital care [31]. These quality indicators merely focus on clinical care processes and were developed in a collaborative process with the inspectorate, hospital federations, and medical specialist and nursing societies [32]. Furthermore, financial and administrative information, information from calamity reports and earlier visits, and judgments of the inspectorate provide input for risk detection.

Although research shows that IGZ inspectors expect patients to be capable of detecting poor performance or risks that might be missed by regular inspection visits [29], patient’s experiences are not yet included, systematically, in Dutch risk detection [33]. However, the inspectorate has become more interested in using information from rating sites to expand their methods to detect poor performance [34] having been stimulated by their colleagues working in health care supervision abroad and by the growing emphasis on patient participation [29]. In addition, an earlier study had already shown that the largest rating site in the Netherlands, ZorgkaartNederland, appeared to be the only social media source that was of additional value for risk-based supervision of elderly care [26]. Using rating sites by the Dutch health care inspectorate to detect poor quality of care could be an important development in several ways. First, the IGZ wants to involve the patient’s perspective in supervision, as the inspectorate’s primary client is the citizen [35]. The inspectorate therefore needs reliable sources that express the patient’s perspective on quality and safety of health care. Second, stakeholders, such as the health care inspectorate, may give patients a voice by using rating sites, which may encourage them to share their experiences. Besides, it may stimulate health care providers to improve their quality of care, knowing that both patients and stakeholders take these rating sites seriously.

The aim of our study was therefore to explore whether and how patient experiences reported on rating sites can, in the eyes of health care inspectors, contribute to risk identification in hospital care.

We address 3 research questions:

1. Do health care inspectors already use patient experiences on rating sites in their daily supervision of hospitals and in what way?

2. Do inspectors expect patient experiences in hospitals, reported on rating sites, to contribute to their estimation of risk?

3. Does presenting, actively, patient reviews reported on the rating site ZorgkaartNederland alert inspectors in their estimation of risks to patient safety?

## Methods

Given the unexplored nature of the topic, an exploratory, interview study was designed.

We used a semistructured interview approach along with an investigation of the judgment of the review data from a patient rating site. The consolidated criteria for reporting qualitative research (COREQ) guidelines [36] were followed to ensure the completeness of the reporting.

### Sample

For the supervision of hospital care, the IGZ divided the field into 10 segments. Each segment covers 10 hospitals on average with 1 senior inspector being responsible. Our sample thus consisted of 10 senior inspectors.

### Study Design and Procedure

#### Step 1: First Round of Interviews, Exploring Use and Views

In January and February 2015, the primary researcher (SK) performed the first round of semistructured interviews with the senior inspectors to establish their actual use in the supervision of health care, of patient experiences reported through rating sites and to explore their views on the potential contribution of such patient ratings (research questions 1 and 2). They were approached by email. The interview guide consisted of general topics concerning attitude to social media in general for working and private purposes; use of patient rating sites for working purposes; and (expected) value of the use of rating sites for supervision. These general topics consisted of several open questions, which were merely explorative: “What do you think of… and why?” Interviews were recorded on audiotape. Field notes were made during the interviews. The interviews lasted up to 1 hour. The first 2 interviews were discussed with 2 researchers (IB and RK) to ensure completeness and interview techniques.

#### Step 2: Selecting Hospitals and Reviews

After the first round of interviews, the inspectors were provided with texts of negative reviews on the rating site ZorgkaartNederland regarding one of the hospitals under their supervision. ZorgkaartNederland [37] is the Federation of Patient and Consumer Organizations’ (NPCF) noncommercial patient rating site [38]. It has the largest number of patient ratings in the Netherlands, with more than 300,000 ratings in total and 800,000 unique visitors per month. Patients can anonymously rate either the care organization or their care provider on a scale of 1 to 10 based on 6 factors: appointments, accommodation, employees, listening, information, and treatment. The average of the 6 scores yields the overall rating, which is a valid summary of the factor’s scores [39]. Patients have to clarify their rating with a written review checked by the website’s editorial office. This helps to mediate the risk of unfounded ratings. Even so, the editorial office checks the internet protocol address of every individual review, thus generating information on whether a patient has provided multiple ratings, which could be used to filter out ratings that appear to be duplicates. Patient characteristics are not asked for, so case-mix correction is not possible [40].

We defined a rating as a quantitative score given to a hospital or doctor and a review as a written comment [18]. For each inspector we selected, at random, 1 hospital under their supervision. Only hospitals with at least 50 ratings in the period from November 1, 2013 until October 31, 2014 (1 year) were eligible, to have a substantial number of ratings. Besides, at least 10 negative ratings had to be available for this hospital, as the reviews belonging to these ratings were expected to contain most useful information for inspectorates [26]. Therefore, we categorized the average overall rating using a classification derived from the international known measure of recommendation, the Net Promoter Score. This measure considers the numbers 9 and 10 as positive (“promoters”), the numbers 7 and 8 as neutral, and the numbers 0 till 6 as negative recommendations (“detractors”) [41]. If the hospital had less than 10 negative ratings, we selected, at random, another hospital. The hospitals selected had on average 21 negative ratings (see Table 1).

**Table 1:** Rating overview of the hospitals selected and of all the hospitals covered by ZorgkaartNederland (November 1, 2013-31, October 2014).

	Ratings of 10 selected hospitals (mean of the 10 hospitals (range))	Ratings of all (94) hospitals on ZorgkaartNederland (mean of the 94 hospitals (range))
Total number of ratings	129 (65-170)	173 (4-859)
Mean rating score^a^	8.2 (7.9-8.6)	8.5 (7.5-9.1)
Positive ratings (score>8.4)	86 (40-116)	122 (3-598)
Neutral ratings (score 6.5-8.4)	22 (11-33)	36 (1-250)
Negative ratings (score<6.5)	21 (12-28)	15 (0-56)
Percentage >6.4	83.6 (78.5-89.7)	91.1 (67.9-100)
Percentage <6.5	16.4 (10.3-21.5)	8.9 (0-32.1)

^a^ Rating score: average of 6 scores on a scale of 1 to 10 regarding appointments, accommodation, employees, listening, information, and treatment.

Subsequently, we presented the texts of the negative reviews of the hospital selected in an Excel sheet, which was sent by email to the inspectors. We also provided the hospital’s contextual information such as the name, the mean rating, the total number of positive and negative ratings, and the percentage of negative ratings, as compared with other hospitals, and what level the review was attributed to: hospital, location, department, or doctor. Inspectors were asked to score the relevance of each negative review for the health care inspectorate according to a previously developed ordinal assessment scheme [26]: “no additional value (0),” “relevant, information leads to a signal in the file of the organization (1),” “relevant, information leads to further investigations (2),” or “relevant, information leads to immediate action (3).” We choose to reveal the name of the selected hospital to explore whether inspectors would find out new information or merely information that was supportive of what they already knew from their experiences with the hospital. Inspectors filled in the score list and returned it to the researcher before the second interview.

#### Step 3: Scoring Negative Reviews and Identifying Underlying Motives

The primary researcher (SK) performed a second round of interviews from April until June 2015. The aim was to determine whether the reviews contained information on risks to patient safety (research question 3). These interviews consisted of 2 parts. In part 1, inspectors were queried about their judgment of each negative review and were asked what elements in the text of the reviews triggered their scoring. We provided some possible triggers, such as the subject, the tone, the concreteness, or the extensiveness of the review. In addition to these, the inspectors could always add new triggers. In part 2, inspectors were asked their general opinion about the use and value of the judged reviews for daily supervision work. The topic list included items such as usability, reliability, new or known information, and value for risk estimation. These interviews were also tape-recorded.

### Analysis

All interviews were transcribed verbatim and were sent to the interviewees for triangulation.

They were analyzed following guidelines for qualitative research [42,43] and by using a digital qualitative data analysis program, Atlas-ti [44]. Analysis was performed in parallel with the interviewing. In the first round of interviews, the first interviews were analyzed inductively, aiming to explore and identify relevant views and propositions. In the second round of interviews, open coding (summarizing and categorizing the data) was gradually replaced with axial coding (confirmation of codes and the identification of broader relationships). Finally, data were clustered across interviews to derive common themes. The inspector’s scoring of the negative reviews was analyzed descriptively [26]. The arguments for the scores were described. We performed a member check by sending all quotes to all interviewees to ensure interpretation and hereby validity.

## Results

### Sample

All 10 senior inspectors consented to participate in both interviews. Their average age was 53 years (range 40-64). Seven were women. All inspectors were educated as a health care professional and had worked in a hospital for several years. The average number of working years as an inspector was 8.5 years (range 1-17). Four inspectors used social media (Twitter, Facebook) for private purposes. All used the Internet for their work (Google, ZorgkaartNederland, Twitter, news websites).

### Inspector’s Current Use of Patient Rating Sites in Daily Supervision

The first round of interviews addressed the first research question, whether health care inspectors already used patient experiences on rating sites in their daily supervision of hospitals and in what way.

Seven inspectors used ZorgkaartNederland to gather information in their supervision work. When preparing their annual meeting with the board of a hospital or in case of reports of serious incidents, they looked for information on search machines such as Google and then ended up at the patient rating site ZorgkaartNederland.

Then I google that person. You end up at ZorgkaartNederland very quickly. The first hit of Google apparently is ZorgkaartNederland.

Respondent 3

In particular I use ZorgkaartNederland, in any case I look at it in preparation for the annual board interview. And, if we focus on a specific doctor involved in a report or for example because of the suspicion of incompetence, then I check ZorgkaartNederland for the individual judgment relating to the doctor.

Respondent 2

Three inspectors did not use the patient rating site, ZorgkaartNederland. They did, however, gather their information from the Internet, but in their cases from hospital websites, newsletters, or news websites, not from a source that contains the patient’s perspective.

I read newsletters from hospitals. (…) But Twitter is also a possible source. (…) For me that is easy to read, and very handy because I can scan very quickly whether it is valuable for me or not.

Respondent 6

### Inspector’s Anticipated Value of Patient Rating Sites for Daily Supervision

The first round of interviews also addressed the second research question, whether health care inspectors expect patient experiences in hospitals, reported on rating sites, to contribute to their estimation of risk to patient safety.

All inspectors who ended up at ZorgkaartNederland indicated that they find it hard to use this information or give weight to this information in their daily supervision.

I think you should be very careful with this information. It must be seen as a signal, not more than that. A signal deserves to be taken seriously and to be properly checked and verified.

Respondent 10

What do you do with it? You take it with you. In that way you use it, but concretely in the conversation with the hospital board, or, in the reports, no, you do not use it that way.

Respondent 1

Thus, apart from a source for gathering information, the 7 inspectors using ZorgkaartNederland did not apply the content of the information for risk identification in their daily supervision practice. However, they saw the reviews as a signal, providing interesting background or contextual information. In the opinion of 5 inspectors, these signals should always be verified and checked by other available information.

In fact it is an indicator. An indicator always needs further research. It must be seen in combination with other indicators: what are the connections and the relevant themes?

Respondent 2

The inspectors brought up 3 main doubts concerning the weight and value of ZorgkaartNederland as a source for identifying risks. Firstly, 4 inspectors feared bias or selectivity, that is they felt that only a small group of people uses rating sites.

The number of reviews is too small to be taken seriously. Only a small group of patients makes the effort.

Respondent 5

Inspectors felt that this group is probably not representative of the patient population of a hospital. For example, hospitals might stimulate very satisfied patients to rate their experiences, to raise their average rating. Besides, positive reviews may have been posted by family and friends of the doctor. Second, 9 inspectors indicated that reviews are often too subjective and emotionally driven. Accordingly, reviews may polarize public opinion at a certain moment and can be used for unnecessarily blaming the doctor.

I feel the psychology of reviewers on a rating site is interesting. In fact, there is a lot of psychology on those sites. People parrot each other easily and therefore strengthen the message and are thus polarizing what happened at a certain moment. And that gives an incorrect picture of the hospital or doctor. It is influenced too much by the moment and the polarization. We should be aware of that.

Respondent 8

It can be used for blaming and shaming. That is very easy on the Internet because it is safe and anonymous.

Respondent 9

Third, inspectors had doubts about the relevance of the content of reviews for the inspectorate’s estimation of risk. Negative reviews were thought to contain mostly remarks on the way patients are addressed, the bad food, signage, or waiting times, not about potential risks to safety.

Patients talk on a very basic level, often about how patients are addressed, and that is not within our remit.

Respondent 5

I do not know how to interpret the reviews. You know, if a doctor is nice he gets an eight although technically speaking he is not so good. The patient cannot interpret that. (…) I feel that is no use for me.

Respondent 6

If information on ZorgkaartNederland could be integrated into other sources of information on patient safety, most inspectors would consider this information to contribute toward the identification of risks. They indicated that the value of reviews for their supervision would improve if the reviews were supported by facts and were substantial but also that the tone of the texts matters.

It depends on whether the review is supported by facts. If it is written in concrete, correct sentences (…) I would rather adopt it than when it is a story of verbal abuse like “it was really awful” with a lot of emotions.

Respondent 10

Yet, the inspectors indicated that they would be triggered to act if a review contains medical errors, serious incidents, damage, unacceptable care or, shortcomings of care. Those reviews would be taken more seriously than reviews about how patients are addressed or about complaints. They would also pay attention when the number of negative reviews suddenly rises because this could be a signal of failing. The inspector should have the feeling that the review was not written impulsively,

“but that another reasonable patient could echo this judgment as well.

Respondent 8

### Experienced Relevance of Patient Rating Sites for Daily Supervision

The second round of interviews addressed the third research question whether actively presenting patient reviews reported on the rating site ZorgkaartNederland alerts inspectors in their estimation of risks to patient safety.

In total, 207 negative reviews were presented to the inspectors, who scored these according to their relevance. The inspectors scored 47 (22.7%) reviews “relevant” (score 1, 2, or 3; see Table 2).

**Table 2:** The relevance of reviews as scored by the inspectors.

	Negative reviews (N)	Percentage	Percentage of “relevant” scores
No additional value (0)	160 (in 10 hospitals)	77.3	—
Relevant, information leads to a signal in the file of the organization (1)	31 (in 7 hospitals)	15.0	66.0
Relevant, information leads to further investigations (2)	15 (in 6 hospitals)	7.2	31.9
Relevant, information leads to immediate action (3)	1 (in 1 hospital)	0.5	2.1
Total	207	100	100

#### The Reasons Reviews Were Considered Irrelevant

Most of the reviews that were scored as nonrelevant for supervision (160/207) were labeled as a complaint dealing with how patients were addressed, the attitude of the doctor, information and communication, or waiting times. Inspectors indicated that dealing with such complaints is a task of the hospital itself, that is, the board or a complaint officer or committee.

This is about how the patient is addressed such as bad experiences with being listened to. I reckon that this happens in every hospital and I am convinced that a lot of improvements can be made in this respect, but it is not a task of the health care inspectorate.

Respondent 7

Other motives not to score the review as relevant were their vagueness, the shortness of the description, or the highly emotional tone such as with comments like:

"He is a horrible man." That man may well be horrible, but what can the health care inspectorate do about it?

Respondent 3

#### The Reasons Reviews Were Considered Relevant

Thirty-one reviews (31/207; 15%) were scored as “relevant, information leads to a signal in the file of the organization” (score 1). The reasons why inspectors gave this score were:

- The review mentioned risks concerning quality and safety.
- The review had a medical content.
- The review could indicate a structural problem, such as shortcomings in care for vulnerable elderly patients or children; therefore, it could contribute to the compilation of a file on that particular hospital or department.
- The doctor was also an instructor to students.
- The department or doctor were well-known, for instance from an earlier investigation, or an underperforming department.

I know this doctor, he came up more often in conversations. He is also mentioned in an earlier investigation. Although no serious incidents have been reported against him, he is known to be a difficult man to deal with—so to speak!

Respondent 1

Fifteen reviews (15/207; 7.2%) were scored as “relevant, information leads to further investigations” (score 2). The reasons the inspectors gave for considering these reviews to be of greater relevance were medical, procedural, or related to the hospital’s profile:

- The review mentioned serious incidents or surgical or medical errors, complications, or damage to the patient or other major consequences such as a long length of stay; or the review concerned medication, it was, for instance, forgotten, or a prescription meant for another patient was given in error during discharge from the hospital.
- The review concerned actual procedural themes in the hospital, for instance, deficiencies in procedures concerning the primary treating physician, about cardiac rehabilitation, or about shortcomings with anticoagulants.

If reviews concerned the hospital’s profile, this might indicate 2 possibilities. Either the review was about a topic in which the hospital was not specialized;

This hospital has no department for genetic research, so in that context, if genetic factors play a role, it should be taken care of by specific procedures. And, according to this review there was insufficient attention given to genetic factors.

Respondent 8

Or the review was related to a topic in which the hospital was specialized.

This hospital is a bariatric center. Given that context this should not have happened here.

Respondent 2

One review (1/207; 0.5%) was indicated as “relevant, information leads to immediate action” (score 3). The considerations given by the inspector were:

- The review described a serious incident, which was also reported to the inspectorate.
- The review concerned an already notorious doctor.
- Moreover, the hospital had not reacted properly after this serious incident.

#### Additional Considerations Regarding Relevance of Reviews

The inspectors mentioned several other considerations for judging reviews to be of greater relevance:

- the number of reviews concerning a specific department, doctor, or topic;
- the concreteness of the review;

Five operations, two times outpatient operations, five infections; these are concrete facts which make me wonder what kind of operation room was that?

Respondent 4

- their own opinion and experiences with how the hospital was functioning;

I am aware of a serious incident that happened recently in this department, so when I saw this review I was alerted. Then I saw another review about a doctor and again it was this same department. So maybe there is more going on there.

Respondent 3

- the given period of time and the actual events that took place in the hospital;

This hospital has had a lot of negative publicity in that specific period. I think that is reflected in the negative reviews.

Respondent 7

- the ranking of the hospital on other well-known ranking lists;

Since several years this hospital is on top of a number or ranking lists. However, last year it fell down (…) I think it is interesting to interpret this period, especially where does this organization come from, where are they now and where are they heading for?

Respondent 2

- what was already known by the IGZ from other quality indicators;
- the contextual information about the mean scores of all hospitals was considered by most inspectors in their assessment of the reviews as valuable, but never decisive.

The percentage of negative reviews is high compared to other hospitals, but maybe this hospital challenges patients to offer a rating on ZorgkaartNederland. That fits in with the positive picture I have of this hospital.

Respondent 3

### New Information or Already Known?

The actions of 9 inspectors were triggered especially by reviews that confirmed their knowledge about, and experience with, the hospital. In these instances, the reviews on ZorgkaartNederland supported the other sources of information used. Five inspectors explicitly indicated that the reviews rendered new information, mostly concerning a specific doctor or department that was mentioned more than once in the reviews.

For me it resulted in two new points of attention: this doctor, who was mentioned four times and I have never heard of, and also the critical remarks about that specific department I did not know of.

Respondent 5

In summary, after having been confronted with the reviews, the inspectors mentioned 2 ways in which they could use this information from ZorgkaartNederland in future supervisory work. According to 9 inspectors, this information could be used to put topics, departments, or specific doctors onto the agenda in the yearly interview with a hospital board.

I would mention it as a signal: I saw on ZorgkaartNederland that...Have you seen it as well and what do you think about it? And if so, what have you done about it?

Respondent 10

Three inspectors indicated that this new information could be used in unannounced visits to the hospital, especially referring to specific departments who came to attention through the reviews.

We assess a lot of things, indicators, reports of serious incidents, but if you look for themes in order to make an unannounced visit, this could be part of it, definitely. People make an effort to write a review on ZorgkaartNederland, they do that on purpose.

Respondent 9

## Discussion

We examined whether and how patient experiences as reported on patient rating sites have a potential to contribute to hospital inspectors identification of risks to safety. Currently, most inspectors only use patient experiences on the patient rating site, ZorgkaartNederland, as a source for gathering background or contextual information about a hospital or a doctor. It automatically arises with searching the Internet. However, for most inspectors, this appears to lead to the question: what exactly to do with the ratings and reviews and how to determine the value of the picture they get? This could be caused by 3 main objections brought up by the inspectors at the beginning of this study. First, inspectors worry about how representative the patient rating sites are, given, for instance, the selected group of patients responding and the relatively low number of ratings. Second, they indicate that reviews are often too subjective and emotionally driven. Third, they had doubts about the relevance of the content of these reviews for supervision.

Earlier research showed, too, another objection among inspectors to the use of patient rating sites for supervision. This was their concern about whether patients are able to evaluate the medical expertise and capabilities of an individual doctor [29,45].

Concerning how far rating sites are representative, it is known from literature that users of patient rating sites significantly differ from nonusers on sociodemographic and psychographic variables and health status. Users are significantly younger and more highly educated. Also, female patients and patients with chronic diseases use patient rating sites more often than other patient groups [17,46]. However, research on the data provided by ZorgkaartNederland [40] showed that the self-selected sample of patients on ZorgkaartNederland did, in fact, lead to representative ratings about Dutch health care in hospitals. Moreover, research into the content of reviews showed that the review process is not just a one-off reflection of a single moment but contextualizes this within a series of previous experiences [45]. This may place the prevalent “n=1” objection in perspective.

The subjectivity of patient’s assessment is a well-known discussion in literature. Indeed, a patient’s assessment of care is subjective, by nature. Nevertheless, a lot of research has been done, showing positive relationships between patient’s (subjective) assessments and the quality of care, patient safety, and clinical effectiveness [47-53].

Although there is evidence of the correlation between scores on patient rating sites and quality indicators and clinical outcomes on a hospital level [5,8,19-21], little research has been carried out on the association between patient ratings and physician quality metrics. Gao et al found a significant positive relationship between Web-based ratings and physician quality as shown by board certification, education, and malpractice claims [8]. However, more research on this topic is needed to overcome this objection.

Despite their reservations regarding the use of patient rating sites for daily supervision, when confronted with the text of negative reviews from one of the hospitals under their supervision, inspectors scored 23% of the reviews as being relevant for risk estimation. Reviews were indicated as relevant when they contained information about major safety problems such as medication errors, serious incidents, severe damage or consequences for the patient, structural organizational problems such as a malfunctioning department or doctor, actual themes, and whether the reviews are in line with the hospital’s profile. Many of these “medical” indicators of possible relevance were also mentioned by inspectors at the beginning of the study, before having scored the reviews presented. However, the scoring of the reviews also revealed new relevant indicators such as structural and procedural organizational problems, which could produce a relevant score for risk estimation.

Compared to previous research carried out on reviews from ZorgkaartNederland concerning their additional value for supervision in the long-term elderly care [26], the percentage of reviews considered relevant by hospital inspectors was lower (23% vs 62%). However, from the relevant hospital reviews, 34% is seen as “relevant, information leads to further investigations” (score 2) or even “relevant, information leads to immediate action” (score 3), compared to 15% in the elderly care. As compared with long-term elderly care, safety issues in hospitals might be judged as being serious at an earlier stage, given the high-risk processes involved. The high number of reviews judged to identify safety issues is in line with patient safety literature, which states that there is evidence to suggest that hospital patients can be used as partners in identifying poor and unsafe practice and help enhance effectiveness and safety [48,54]. Although most comments are classified as physician-related concerns [4,14], content analyses of reviews in literature showed 3 dominant themes indicated by patients: interpersonal manner, technical competence, and system issues. These all include potential risks to patient safety [14]. It is important to note that the use of rating sites is likely to increase in the near future when the generation socialized with social media reaches the age in which health questions and doctors become dominant. As a result, these kinds of sources might become even more relevant [3,4,12,46] for patients and physicians, as well as for stakeholders such as the health care inspectorate.

### Implications and Future Research

Hospital inspectors at first showed some restraint in their concrete use of ZorgkaartNederland in their daily supervision. However, after being confronted, the negative reviews of one of the hospitals under their supervision, most inspectors were cautiously positive about the contribution of the reviews to their risk identification. Nevertheless, they insisted that the use of rating sites should always be accompanied and verified by clinical indicators. The caution of inspectors for the use of reviews from patients is a point of concern for supervision policy in the near future. It appears to be worthwhile to provide health care inspectors regularly with a summary of negative reviews on carefully edited rating sites such as ZorgkaartNederland, complemented with contextual information, regarding hospitals under their supervision. Almost all inspectors indicated that specific themes, departments, or doctors on ZorgkaartNederland could be presented in their annual interview with the hospital board. Also, specific departments that showed up negatively in the reviews could be subjected to unannounced visits. However, evaluating the value and usability of this additional source for hospital supervision in the near future is necessary. Furthermore, it takes more research to understand and support the additional value of the patient’s perspective on quality of health care, for instance, by comparing the patient’s perspective with clinical outcome indicators or with supervision judgements.

A positive aspect of using ratings and reviews in supervision is the availability of actual information, in addition to the yearly available conventional quality indicators. Thus, a more efficient way of risk-based prioritizing within a huge number of health care organizations is a possibility [26]. This is especially important in health care sectors with a substantial number of organizations or professionals such as the elderly care sector, general practitioners, dentists, and pharmacists. In this way, patient ratings and reviews can become a structural part of the supervisory framework for risk detection.

However, most of the ratings on ZorgkaartNederland are positive, as is the case for most rating sites [4,10,11,17,18]. Furthermore, the percentage of negative ratings is decreasing in time, from 19.9% in 2010 to 7.2% in 2015 [55]. This may implicate that poor performance cannot be exclusively depicted by rating sites. Preferably, information from rating sites should be accompanied by other sources to express the patient’s perspective, such as general patient experiences or satisfaction surveys. Furthermore, patients could be stimulated by the government, hospitals, health care providers, and patient organizations to place their experiences on rating sites such as ZorgkaartNederland, to cover a more broad spectrum of patient experiences. For example, the branch organization of long-term elderly care and the NPCF, as owner of ZorgkaartNederland, organizes so-called road teams since 2015. These teams visit institutions of elderly care with mobile devices connected to the elderly care section of ZorgkaartNederland, interviewing clients and relatives to increase the number of ratings substantially. Moreover, since July 1, 2014 a National Reporting Centre for Health Care Complaints (Landelijk Meldpunt Zorg) in the Netherlands gives patients and relatives an opportunity to express their complaints about care, always after having first complained at their provider. The health care inspectorate is given insight in these complaints and can use this information as additional source to detect poor performance from the patient’s perspective.

### Strength and Limitations

This study has strengths and also limitations. The fact that the patient rating site ZorgkaartNederland is an independent, noncommercial website, with its own editorial office that judges the reviews one by one on their substantiating text and checks on the sender of the rating, is a strength of this patient rating site. It increases the value of the reviews. This is not necessarily the case with all patient rating sites in other countries.

The hospitals selected were not necessarily representative of hospitals on ZorgkaartNederland. However, the focus of our research was on the identification of risks in the texts of the negative reviews. Therefore, we wanted a substantial number of negative reviews per hospital and put the minimum threshold on 10. In that way, it was possible to identify trends, themes, departments, or doctors that were, for instance, mentioned more than once.

In this research design, we selected, for each inspector, a hospital for which he or she was responsible. In fact, most inspectors have known these hospitals for some time. They therefore assess the reviews according to their own point of reference, consisting of their accumulated knowledge and experiences. This can be a support to information already known by the inspectors, for instance, about a dysfunctional department. However, this could also blind the inspector to new insights or safety aspects. It would be worthwhile to investigate, in a future study, whether an inspector unacquainted with a certain hospital, would come to the same or a different selection of relevant reviews.

Furthermore, this is a case study among hospital inspectors in the Dutch health care setting, and more research in other settings is needed to draw general conclusions about the usability of patient rating sites for risk detection in supervision.

### Conclusions

Patient rating sites may contribute to the risk-based supervision of hospital care of a health care inspectorate. Health care inspectors do have several objections against the use of patient rating sites for daily supervision. However, when they are presented with texts of negative reviews from a hospital under their supervision, it appears that most inspectors consider it as an additional source of information from the patient’s perspective to detect poor quality of care. Still, it should always be accompanied and verified by other quality and safety indicators. Preferably, it should also be accompanied by other methods to reveal patient’s experiences, to broaden the patient’s perspective on quality and safety of care. Furthermore, more research on the value and usability of patient rating sites in daily hospital supervision and other health care settings is needed.

## Abbreviations

IGZ: The Dutch health care inspectorate
NPCF: Federation of Patient and Consumer Organizations
